# Tumor necrosis factor alpha inhibits ovulation and induces granulosa cell death in rat ovaries

**DOI:** 10.1007/s12522-014-0201-5

**Published:** 2014-12-13

**Authors:** Yuri Yamamoto, Akira Kuwahara, Yuka Taniguchi, Mikio Yamasaki, Yu Tanaka, Yukari Mukai, Mizuho Yamashita, Toshiya Matsuzaki, Toshiyuki Yasui, Minoru Irahara

**Affiliations:** ^1^ Department of Obstetrics and Gynecology The University of Tokushima, Institute for Health Biosciences 3‐18‐15 Kuramoto‐cho 770‐8503 Tokushima Japan

**Keywords:** Apoptosis, Autophagy, Granulosa cell death, Ovulation, TNF‐alpha

## Abstract

**Purpose:**

We evaluated the role of tumor necrosis factor alpha (TNFα) in rat ovulation and granulosa cell death of ovarian follicles during the periovulatory stage.

**Methods:**

Immature rats primed with pregnant mare serum gonadotropin were injected intraperitoneally with human chorionic gonadotropin (hCG), and TNFα was injected into the bursa 48 h later. The total number of released oocytes was counted. Apoptosis was measured with terminal deoxynucleotidyl transferase‐mediated dUTP nick end labeling (TUNEL) and the expression of cleaved caspase 3 and Bax/Bcl‐2. Autophagy was assessed by the expression of light chain protein 3 (LC3) and autophagosomes under transmission electron microscopy.

**Results:**

TNFα significantly decreased the number of released oocytes, and many unruptured follicles were observed. TUNEL analysis revealed a larger number of apoptotic cells, and the cleaved caspase 3 and Bax/Bcl‐2 increased more than that of the control 12 h after hCG administration. Furthermore, the expression of LC3 wwas significantly higher than that of the control, and autophagosomes were observed in the cytoplasm.

**Conclusions:**

Our data indicated that TNFα is an important mediator of ovulation in terms of decreasing the number of released oocytes and inducing granulosa cell death of unruptured follicles via apoptosis and autophagy for remodeling ovarian tissues.

## Introduction

Tumor necrosis factor alpha (TNFα), a nonglycosylated protein with a molecular weight of 17 kDa, is produced by activated macrophages [1]. TNFα is a multifunctional cytokine and mediates a wide range of biological actions that include not only the regulation of proinflammatory responses but also the control of cell differentiation, tissue renewal, and restructuring [2]. Recent studies have demonstrated that TNFα plays a role in ovarian follicular development [3], steroidogenesis [4, 5], ovulation [5, 6], luteolysis [7], and atresia [8, 9]. TNFα is localized in human oocytes and cumulus granulosa cells from aspirated follicles, and TNFα immunostaining has been observed in oocytes of human primordial follicles [10]. In the rat, TNFα is expressed in oocytes, macrophages, and granulosa cells [11]. Ovulation appears to be a cytokine‐regulated inflammatory process. We have reported that cytokine‐induced neutrophil chemoattractant (CINC/gro), which belongs to the interleukin (IL)‐8 family, acts as a functional chemoattractant for neutrophils in rats, and that IL‐1β and TNFα stimulate the production of CINC/gro protein [12]. Increased TNFα could induce follicle atresia, apoptosis and autophagy, and TNFα is considered to be one of the important factors in the periovulatory stage, however, the actual role of TNFα and its effect in the ovary are not completely understood.

Follicular atresia is initiated within the granulosa layer and, subsequently, in theca cells [13]. Widespread cell loss within the granulosa layer induces follicle death. However, oocyte loss seems to be responsible for follicular degeneration only in primordial and primary follicles [14, 15, 16]. The main type of cell death in granulosa cells is apoptosis [17]. Many apoptosis‐related factors have been implicated, including death ligands and receptors, caspases, Bcl‐2 family members, and gonadotropins [18]. Another type of cell death in the ovary is autophagic cell death. Autophagy involves the sequestration of cytosol or cytoplasmic organelles within double membranes, thus creating autophagosomes that subsequently fuse with lysosomes, thereby forming autophagolysosomes [19]. Recently, autophagy has been shown to involve follicular atresia and luteolysis [20, 21]. The amount of light chain protein 3 (LC3) present shows a good correlation with the number of autophagosomes and is frequently used as an autophagy marker. LC3 is converted from LC3‐I to LC3‐II during autophagy induction.

TNFα has been suggested to affect apoptosis in the ovary. However, the exact mechanism of how TNFα induces granulosa cell death has only been partially characterized.

We evaluated the role of TNFα in rat ovulation and granulosa cell death of ovarian follicles during the periovulatory stage. Apoptosis was assessed with the terminal deoxynucleotidyl transferase‐mediated dUTP nick end labeling (TUNEL) method and by analyzing the mRNA and protein levels of Bcl‐2 (antiapoptotic), Bax (proapoptotic), and cleaved caspase 3. Autophagy was assessed by visualizing autophagosomes uisng electron microscopy and by determining the expression of LC3.

## Materials and methods

### Reagents

Modified human tubal fluid (mHTF) medium was obtained from Irvine Scientific (Santa Ana, CA, USA), and pregnant mare serum gonadotropin (PMSG), human chorionic gonadotropin (hCG), and recombinant rat TNFα were obtained from Sigma Aldrich, Japan.

### Animals and superovulation

All experiments were conducted in accordance with the ethical standards established by the institutional animal care and use committee of the University of Tokushima.

Immature female Wistar rats (21 days old) were obtained from Charles River, Japan (Yokohama, Japan). Superovulation was induced with intraperitoneal injection of 10 IU PMSG followed by 10 IU hCG 48 h later. Along with the hCG injection, 50 ng rat TNFα and 100 ng rat TNFα (TNFα group) or normal saline (control group) were injected into the bursa. In this model, ovulation occurred between 12 and 15 h after hCG administration. Rats were killed by cervical dislocation at 6, 12, 24, or 48 h after hCG administration, and ovaries were excised from each animal. One ovary was immediately bathed in RNA stabilization solution (RNAlater; Ambion, Austin, TX, USA) and stored in RNAlater at −20 °C until use. The other ovary was fixed in 4 % paraformaldehyde at 4 °C overnight and embedded in paraffin.

### Number of oocytes and histological examination of the ovaries

The total number of released oocytes was counted. The rats oviducts were removed 24 h after hCG administration. Ovulated cumulus–oocyte complexes were removed from the oviducts, and the cumulus–corona complex was then removed by soaking in mHTF containing 32 IU/ml hyaluronidase to facilitate counting of the oocytes with stereomicroscopy. Sections of ovaries removed 24 h after hCG administration were examined with hematoxylin and eosin (HE) staining.

### Total RNA extraction and reverse transcription (RT)

Frozen ovaries were cut and weighed. Total RNA was extracted from ovaries using the RNeasy Protect Mini Kit (Qiagen, Hilden, Germany) according to the manufacturer's instructions. Total RNA (400 ng) was reverse transcribed into cDNA using 4 U Omniscript Reverse Transcriptase (Qiagen) according to the manufacturer's instructions.

### Real‐time PCR (RT‐PCR) analysis

The primer sequences were designed according to cDNA sequences from Genbank. All primers were synthesized by Sigma Aldrich (Japan). Their sequences and the expected sizes of the PCR products are shown in Table 1. RT‐PCR was performed with Applied Biosystems System Step One Plus (Applied Biosystems, Foster City, CA, USA) using the Fast SYBR Green Master Mix (Applied Biosystems). GAPDH was amplified in parallel in each run as an internal control. The PCR conditions were as follows: 40 cycles of denaturation at 95 °C for 20 s; annealing at 63 °C (Bcl‐2), 64 °C (cleaved caspase 3 and LC3α), 65 °C (GAPDH), 66 °C (Bax), or 68 °C (LC3β) for 30 s; and extension at 72 °C for 60 s. A melting curve was generated at the end of every run to ensure product uniformity (95 °C for 15 s, 60 °C for 60 s, 95 °C for 15 s). The mRNA expression is shown as mean ±SD.

**Table 1:** Forward (For) and reverse (Rev) primer sequences and RT‐PCR product size

	Primer sequence (5′–3′)	Size (bp)
Caspase 3	For CAGAAGCTCCTGCAAAAAGG	
	Rev AGTCTGCAGCTCCTCCACAT	144
LC3‐alpha	For GCCTGTCCTGGATAAGACCA	
	Rev CTTGACTCAGAAGCCGAAGG	241
LC3‐beta	For CAGGTTGCCTAGCAGAGGTC	
	Rev CTCTGAGCAGTGGTGCATGT	181
Bcl‐2	For TTCCAGCCTGAGAGCAACCGAAC	
	Rev TAGCGACGAGAGAAGTCATCCCC	164
Bax	For CAAGAAGCTGAGCGAGTGTCT	
	Rev GGTTCTGATCAGCTCGGGCAC	238
GAPDH	For TGGAGAAGGTGGGGCTCACCTG	
	Rev CCACAACGGATACATTGGGGGTAGGAAC	417

*LC3* light chain 3, *Bax* Bcl‐2‐associated X, *GAPDH* glyceraldehyde‐3‐phosphate dehydrogenase

### Western Blot analysis

The cell lysates (10 μl/lane) were separated on a polyacrylamide gel membrane.

After the nonspecific binding sites were blocked with 3 % skim milk, the membrane was treated overnight with Bax Rabbit monoclonal antibody, Bcl‐2 Rabbit monoclonal antibody and LC3A/B Rabbit monoclonal antibody (diluted 1:1000; Cell Signaling Japan). The immunoreactive bands were demonstrated by incubation with anti‐Rabbit IgG‐HRP (IBL) at room temperature for 1 h. Peroxidase activity was visualized with the enhanced chemiluminescence detection system (Amersham). Integrated optical intensities of the immunoreactive protein bands were quantified by imaging and the analysis software Multi Gauge; they were normalized to GAPDH values.

### In situ end labeling (TUNEL)

DNA fragmentation was analyzed with the TUNEL method using an apoptosis in situ detection kit (TACS2 TdT‐DAB In Situ Apoptosis Detection Kit; Trevigen, Funakoshi, Japan) according to the kit supplier's instructions. For quantifying apoptotic events, cell nuclei (158–234) of five random fields were counted for each treatment. Percentages of the apoptotic nuclei were calculated.

### Transmission electron microscopy

To identify autophagic vacuoles at the ultrastructural level, granulosa cells were fixed with 2 % glutaraldehyde in 0.1 M phosphate buffer at 4 °C, rinsed in phosphate buffer, postfixed in 2 % OsO_4_ in phosphate buffer, dehydrated, and embedded in Epon. Ultrathin sections were contrast stained with uranyl acetate and photographed with a transmission electron microscope (JEM 1200EX; JEOL, Tokyo, Japan).

### Statistical analysis

Variations between groups were analyzed with one‐way ANOVA, followed by Dunnett's multiple range test. Data are shown as the mean ±SD, and a *p‐*value < 0.05 was considered statistically significant.

## Results

### Number of oocytes and histological examination of the ovaries

**Figure 1:**
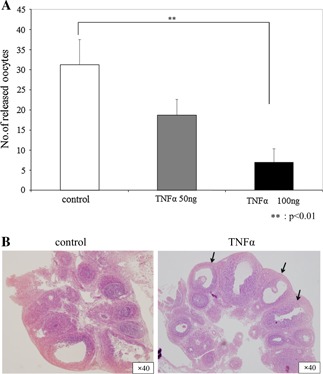
Number of released oocytes and histological examination of the ovaries. The number of oocytes is shown as the mean ±SD (**a**). A significant difference (*p* < 0.01) was found between groups (*n* = 4). Many unluteinized, unruptured follicles were observed 24 h after hCG treatment in the TNFα group (*arrows*), though many corpus luteinized follicles were observed in the control group (**b**)

Twenty‐four hours after hCG administration, the total number of released oocytes decreased in the TNFα group concentration‐dependency (control: 31.3 ±6.2 vs. TNFα50 ng: 18.8 ±3.9 vs. TNFα100 ng: 7.0 ±3.7). Especially, TNFα100 ng was significantly lower than that in the control group (*p* < 0.01; Fig. 1a) (*n* = 4).

Histological analysis revealed normal development of intraovarian follicles; however, follicular rupture was effectively eliminated in the TNFα group. On the other hand, many corpus luteinized follicles were observed in the control group at 24 h after hCG administration (Fig. 1b).

Experiments conducted comparing the TNF100 ng group and the control group included the following:

### mRNA expression

**Figure 2:**
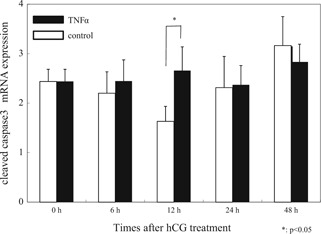
mRNA expression of cleaved caspase 3. Cleaved caspase 3 was significantly increased 12 h after hCG administration compared with the control group (*p* < 0.05). The mRNA expression of each gene was normalized to GAPDH expression, and values shown are mRNA/GAPDH mRNA ratios

**Figure 3:**
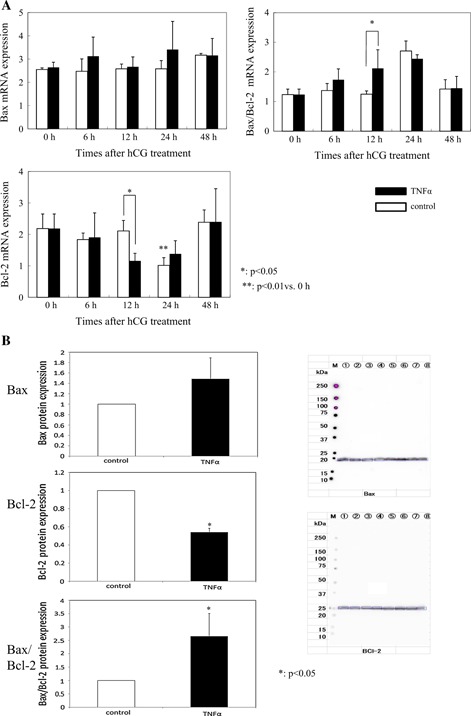
Expression of the Bcl‐2 family. TNFα did not affect Bax expression. TNFα significantly reduced Bcl‐2 mRNA expression (*p* < 0.05), and the Bax/Bcl‐2 mRNA ratio increased (*p* < 0.05) 12 h after hCG administration compared with the control group (**a**). Western Blot analysis at 12 h after hCG administration shows that TNFα significantly reduced Bcl‐2 expression (*p* < 0.01) and the Bax/Bcl‐2 ratio increased (*p* < 0.05) (**b**). *Lanes 1*–*4* were the TNFα group and *lanes 5*–*8* were the control group. They were normalized to GAPDH values

**Figure 4:**
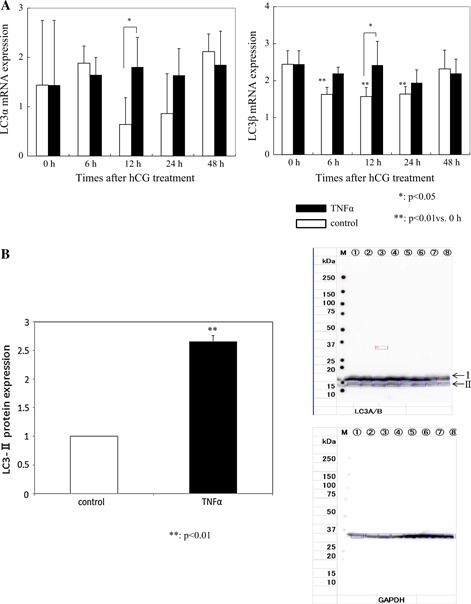
LC3 expression. LC3α and LC3β mRNA expressions were significantly higher in the TNFα group (*p* < 0.05) (**a**), and TNFα also significantly increased LC3‐II protein expression (*p* < 0.01) (**b**) 12 h after hCG administration. *Lanes 1*–*4* were the TNFα group and *lanes 5*–*8* were the control group. They were normalized to GAPDH values

Cleaved caspase 3, which is believed to be the final executioner in apoptotic cell death, was significantly increased 12 h after hCG administration compared with the control group (control 1.80 ±0.50 vs. TNFα 2.49 ±0.48, *p* < 0.05; Fig. 2). TNFα did not affect Bax expression. TNFα significantly reduced Bcl‐2 mRNA expression (control 2.11 ±0.33 vs. TNFα 1.16 ±0.24, *p* < 0.05). The Bax/Bcl‐2 mRNA ratio increased (control 1.25 ±0.11 vs. TNFα 2.12 ±0.33, *p* < 0.05) 12 h after hCG administration compared with the control group (Fig. 3a). At that point, LC3α and β mRNA expression, which correlates with the number of autophagosomes, was significantly higher in the TNFα group (LC3α; control 0.64 ±0.54 vs. TNFα 1.80 ±0.60, LC3β; control 1.57 ±0.24 vs. TNFα 2.42 ±0.64, *p* < 0.05; Fig. 4a) (*n* = 5).

### Western Blot analysis

In order to ensure the PCR results with more certainly, Western Blot analysis and relative determination of protein concentrations were performed 12 h after hCG medication (*n* = 4). Western Blot analysis presented similar results as those of RT‐PCR. TNFα decreased Bcl‐2 expression (0.54 ±4.3, the ratio with the control, *p* < 0.01; Fig. 3b), increased the Bax/Bcl‐2 ratio (2.65 ±0.85, the ratio with the control, *p* < 0.05; Fig. 3b), and increased LC3‐II expression (2.65 ±0.10, the ratio with the control *p* < 0.01; Fig. 4b).

### TUNEL assay

**Figure 5:**
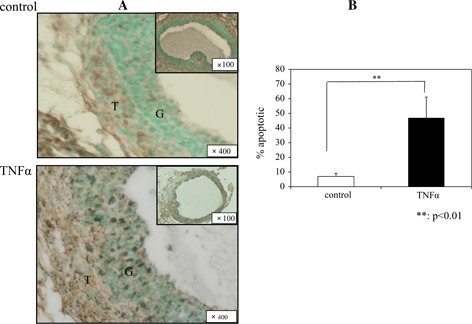
TUNEL staining 12 h after hCG treatment and rate of apoptosis. Apoptotic cells were detected by TUNEL‐positive cells (brown staining). Many apoptotic cells were seen in the granulosa of the TNFα group compared with the control group (**a**). The rate of apoptotic nuclei was significantly higher in the TNFα group than in the control group (*p* < 0.01) (**b**). *G* granulosa cell, *T* theca cell

Twelve hours after hCG administration, we observed that the majority of the follicles in the ovarian sections had not yet ruptured, although a small number of follicles had progressed to atresia. We, therefore, examined the follicles at time points beginning 12 h after hCG administration. Many TUNEL‐positive granulosa cells were observed in the TNFα group, but not in the control group. The apoptotic nuclei rate was significantly higher in the TNFα group (46.9 ±14.2 %) than in the control group (7.0 ±2.1 %), *p* < 0.01; Fig. 5).

### Transmission electron microscopy

**Figure 6:**
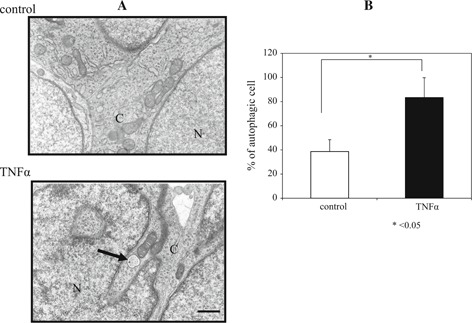
Transmission electron microscopic image of granulosa cells 12 h after hCG treatment. Autophagic vacuoles were present in the cytoplasm in the TNFα group (*arrow*) (**a**). The rate of autophagic vacuoles was significantly higher in the TNFα group than in the control group (*p* < 0.05) (**b**). *N* nucleus, *C* cytoplasm, *bar* 500 nm

Autophagic structures are characterized by multiple autophagic vacuoles, which are double membranous vacuoles. Autophagic vacuoles were seen in granulosa cells in the TNFα group. The proportion of autophagic granulosa cells was higher in the TNFα group (83.3 ±13.4 %) than in the control group (38.6 ±9.8 %), *p* < 0.05; Fig. 6).

## Discussion

In this study, we demonstrated that TNFα decreased the number of released oocytes, and many unluteinized, unruptured follicles were observed 24 h after hCG administration. In the TNFα group, TUNEL analysis showed a larger number of apoptotic granulosa cells 12 h after hCG administration, and the rate of apoptotic nuclei was higher than that of the control. At the same time, expression of cleaved caspase 3, Bax/Bcl‐2, and LC3 were increased substantially more than that of the control. Furthermore, autophagosomes were observed in the cytoplasm of granulosa cells in the TNFα group. Thus, TNFα suppressed ovulation and induced not only apoptosis but also autophagy in granulosa cells. Contrary to our previous study showing that TNFα stimulates the production of CINC/gro protein and promotes ovulation [12], TNFα decreased the number of released oocytes under the condition in this study. According to these data, we believe TNFα plays a very important role during the peri‐ovulatory stage and in regulating ovulatory function. A high TNFα concentration could suppress ovulation.

Previous investigations have demonstrated that TNFα has an inhibitory effect on ovarian steroidogenesis [4, 5]. In addition, it has been suggested that progesterone receptor is required specifically for follicular rupture leading to ovulation, and mice lacking its receptor have lost the ability to ovulate [22, 23, 24]. According to our data, TNFα reduced the number of released oocytes and increased unluteinized, unruptured follicles. Thus, TNFα might control ovulation by suppressing the expression of genes such as StAR or Cyp11a1, which are involved in progesterone production [4]. Further research is needed to reveal the molecular mechanisms.

Apoptosis is thought to induce follicle atresia. Caspases constitute a family of intracellular cysteine proteases that are involved in both the initial and final stages of apoptosis. In particular, cleaved caspase 3, which is expressed in granulosa cells of atretic follicles, plays a central role in the apoptotic program [25]. The Bcl‐2 family of proteins (proapoptic: Bax; antiapoptotic: Bcl‐2) is also implicated in apoptosis [26, 27, 28]. Bax acts as a mitochondrial gateway where a variety of apoptotic signals converge [29]. Bcl‐2 can inhibit Bax‐induced apoptosis by forming heterodimers with Bax [30]. Thus, the Bcl‐2/Bax ratio is considered important for the regulation of cellular apoptosis. Two pathways that induce apoptosis may, thus, be operating. In the first pathway, activated caspase 8 activates executioner caspases. In the second pathway, activated caspase 8 triggers cleavage of the Bcl‐2 family member Bid, which acts on mitochondria, resulting in cytochrome *c* release, caspase 9 activation, and, finally, activation of all executioner caspases [31]. In our experiments, the observation of many TUNEL‐positive granulosa cells, and the increase in the apoptotic nuclei rate, the increase in caspase 3 mRNA, and the decrease in Bcl‐2 revealed that TNFα induces apoptosis in granulosa cells through the caspases and Bcl‐2 family members during the periovulatory stage.

Autophagy was originally thought to represent a survival response to nutrient deprivation. Several reports indicate a role for autophagy in the maintenance of cellular homeostasis [32, 33]. Autophagy involves the creation of autophagosomes or autophagic vacuoles in which cytoplasmic organelles are degraded by lysosomal hydrolases. Recent studies have demonstrated that autophagy may be involved in follicle atresia and luteolysis, and autophagy may be involved in ovarian follicular atresia at all stages of follicular development [18, 19, 20]. LC3 is one of three light chains that form a complex with the microtubule‐associated proteins 1A and 1B. In humans, three genes encode highly homologous LC3 proteins (LC3α, β, and γ), two of which are conserved in rats: LC3α and β. LC3α, β, and γ proteins are either cytosolic (LC3I) or membrane‐associated (LC3II). Because LC3 exists in the outer membrane of autophagolysosomes after hydrolysis, it shows a good correlation with the number of autophagosomes and is frequently used as an autophagy marker. Our results showing that the mRNA levels of LC3α a, β, and LC3‐II protein level increased more than that of the control, and that autophagosomes were observed in the cytoplasm during the periovulatory stage, indicate that TNFα induces not only apoptosis but also autophagy. Thus, TNFα seems to participate in granulosa cell death in unruptured follicles via two types of programmed cell death, apoptosis and autophagy.

According to our data, inflammatory reactions with an increased TNFα concentration in local peritoneum, such as the endometriosis or bacterial infection related adnexitis, could result in over‐expression of TNFα, and induce follicle atresia, apoptosis, and autophagy. It could be interpreted as cells in un‐ovulated follicles vanishing from the ovarian cortex and healthy growing follicles replacing the original area to prepare for the next ovulations. With this function, the ovary could maintain follicular development and ovulation, and at the same time, 99 % of the follicles could go to atresia. It is important to keep the constancy of the ovary to induce un‐ovulated follicular cell death. TNFα may play a part in tissue repair and remodeling of the ovaries by inducing unruptured follicle cell death.

Our data indicated that TNFα plays two roles during the periovulatory period in vivo. One role is to control ovulation in terms of decreasing the number of released oocytes, and the other is to promote unruptured follicle cell death via two courses of apoptosis and autophagy for tissue repair and remodeling of the ovaries.

## Acknowledgments

The authors did not receive any funding associated with this article.

### Conflict of interest

Yuri Yamamoto, Akira Kuwahara, Yuka Taniguchi, Mikio Yamasaki, Yu Tanaka, Toshiya Matsuzaki, Toshiyuki Yasui, Minoru Irahara declare that they have no competing interests.

### Human rights statements and informed consent

This article does not contain any studies with human subjects performed by any of the authors.

### Animal studies

All institutional and national guidelines for the care and use of laboratory animals were followed.
